# Comparison of Effectiveness between Cobalt Chromium Rods versus Titanium Rods for Treatment of Patients with Spinal Deformity: A Systematic Review and Meta-Analysis

**DOI:** 10.1155/2020/8475910

**Published:** 2020-09-01

**Authors:** Frank D. Shega, HongQi Zhang, Daudi R. Manini, MingXing Tang, ShaoHua Liu

**Affiliations:** ^1^Department of Spine Surgery, Xiangya Hospital of Central South University, Changsha City 410008, Hunan Province, China; ^2^Department of Orthopedic Surgery, Mount Meru Regional Referral Hospital, P.O Box 3092, Arusha City, Tanzania; ^3^Department of Orthopedic Surgery, Mwananyamala Regional Referral Hospital, P.O Box 61665, Dar-es-salaam City, Tanzania

## Abstract

**Background:**

Biomechanical properties of rods determine their ability to correct spinal deformity and prevention of postoperative sagittal and coronal changes. The selection of a proper rod material is crucial due to their specific mechanical properties that influence the surgical outcome. The purpose of this study is to compare the effectiveness of cobalt chromium rods versus titanium rods for the treatment of spinal deformity by a systematic review and meta-analysis.

**Methods:**

PubMed, EMBASE, and the Cochrane library were searched for observational and biomechanical studies comparing cobalt chromium and titanium rods in terms of correction rate, thoracic kyphosis, lumbar lordosis, incidence of rod fracture, fatigue life of contoured rod, bending stiffness of rods, and occurrence of proximal junctional kyphosis. The demographic data and mean values of outcomes of interest were extracted from each group and compared by their mean difference as an overall outcome measure. The Review Manager software (RevMan 5.3) was utilized at a 95% significance level.

**Results:**

Eleven eligible studies with 641 participants for 7 observational studies and 35 samples for 4 biomechanical studies were identified. There were no significant differences between cobalt chromium and titanium rods in the correction rate of spinal deformity. Postoperative thoracic kyphosis was well restored in the cobalt chromium group with statistical significance (*p* value = 0.009). The incidence of rod fracture was high in titanium rods compared to cobalt chromium rods with significant difference (*p* value = 0.0001). Proximal junctional kyphosis occurs more in the cobalt chromium group with a significant difference (*p* value = 0.0009). No statistical significance between two materials in terms of lumbar lordosis, fatigue of life, and bending stiffness of rods.

**Conclusion:**

The cobalt chromium rod is better than titanium rod for effective correction of spinal deformity and postoperative stability of the spine. However, the use of cobalt chromium rods is associated with increased risk of proximal junctional kyphosis.

## 1. Introduction

Spinal deformity, one of the commonest orthopedic disorders, is a malformation or malalignment of the vertebral column. It presents as unnatural curvature, as in scoliosis, kyphosis, lordosis, or combined deformities [1]. Apart from idiopathic and congenital etiologies, spinal deformity also occurs due to multiple fractures or ankylosing spondylitis. Its prevalence is reported to increase with age and estimated to be 30%–68% in the elderly population [2]. Pain and disabilities caused by spinal deformities lead to poor health-related quality of life [3].

Management of spinal deformity is based on extensive assessment of the spine with thorough physical examinations, plain radiography, computed tomography scan (CT scan), and magnetic resonance imaging (MRI) to come up with the best treatment plan and determination of prognosis [4]. Posterior spinal instrumentation and fusion using pedicle screws and rigid rods have been a recommended surgical treatment for the correction of spinal deformities in the coronal, sagittal, and axial planes [5–10]. The ultimate aim of corrective surgery in spinal deformities is to prevent progression and to stabilize the spine. The factors influencing postoperative correction outcome include the magnitude of the curve, flexibility of a curve, type of surgical technique, points of fixation, selection of instrumented levels, type of implants used, and postoperative care.

The size of the posterior implant typically determines its biomechanical profile in terms of diameter and material properties. Currently, different types of rods such as stainless-steel rods, titanium alloys rods, and cobalt chromium rods are widely used for the treatment of diverse spine deformities for stability and correction of the spinal deformity. The selection of a proper rod material is critical as each of them possesses different mechanical properties, which are determined by their chemical composition. Cobalt chromium (CoCr) and titanium alloy TA6V (Ti) are mostly used rods as they exhibit favorable biomechanical properties such as resistance to corrosion and compatibility with magnetic resonance imaging (MRI) [11]. However, the titanium has greater elasticity, whereas cobalt chromium has greater stiffness [12]. Contouring of rods plays a significant role in overall postoperative correction outcomes as it can diminish their fatigue strength and increases the risk of rod fracture after implantation [13–15].

Despite the availability of many studies on the surgical outcome of spinal deformities, very few of them focused on the postoperative effectiveness of surgical rods. This study compared cobalt chromium rods with titanium rods, in terms of deformity correction rate, change in thoracic kyphosis angle, change in lumbar lordosis angle, incidence of rod fracture, fatigue life of contoured rod, bending stiffness of rods, and occurrence of proximal junctional kyphosis (PJK), by systematic review and meta-analysis of the available published literature.

## 2. Methods

### 2.1. Eligibility Criteria

Studies comparing the effectiveness of cobalt chromium rods and titanium rods for correction of spinal deformity regardless of age, gender, or follow-up time were eligible for inclusion. Only observational and experimental studies comparing the two groups and has the outcome of interest (correction rate, thoracic kyphosis, lumbar lordosis, incidence of rod fracture, fatigue life of contoured rod, bending stiffness of rods, or occurrence of proximal junctional kyphosis) were eligible for inclusion. Only English and full-text published literature were eligible for inclusion. Studies across the world that abide by the inclusion criteria mentioned above were included.

### 2.2. Information Sources

Three online databases, namely, PubMed, EMBASE, and the Cochrane library, were searched to come up with the list of eligible included studies. No date range customization of searches was set. Corresponding authors of articles searched could be contacted to provide further information or settle unclear explanations. Secondary referencing of eligible studies was done to extend the scope of the searches. The online databases were accessed via the Central South University library's website: http://lib.csu.edu.cn/. The last date of the search was 30th January 2020.

### 2.3. Study Search

To generate a set of citations that are relevant to our study's search question, an advanced search tool was used in all of the three databases previously mentioned. Free text words, as well as MeSH terms, were used to search. Using PubMed, advanced search was customized to “human species” and “full text” as ((((“cobalt”[MeSH Terms] OR “cobalt”[All Fields]) AND (“chromium”[MeSH Terms] OR “chromium”[All Fields]) AND rods[All Fields]) AND ((“titanium”[MeSH Terms] OR “titanium”[All Fields]) AND rods[All Fields])) AND (“scoliosis”[MeSH Terms] OR “scoliosis”[All Fields])) OR (“kyphosis”[MeSH Terms] OR “kyphosis”[All Fields]). The advance search in EMBASE was done by using a combination of keywords (cobalt AND chromium AND titanium AND rods AND scoliosis OR kyphosis) and filtered with “human” and “article or review.” Two authors independently performed the searches: FS and DM. Any differences of thought were settled through discussion with the third and fourth authors: ZQ and TX. Results were exported to computer software known as *Mendeley Desktop,* which was used to manage and keep track of references throughout this study.

### 2.4. Study Selection Process

**Figure 1:**
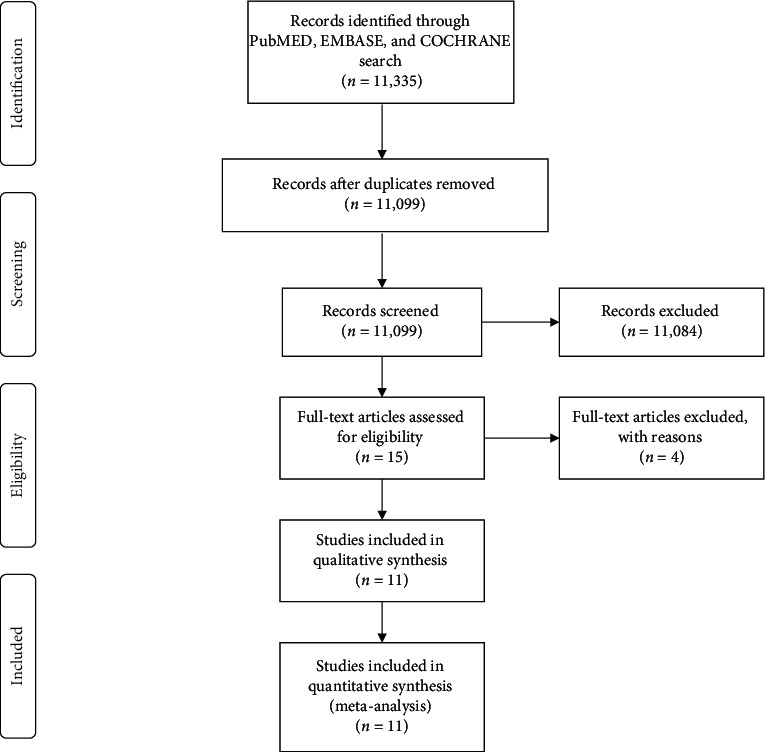
Study selection process according to PRISMA guidelines.

All studies resulting from online database search independently conducted by two authors were initially screened by their titles and abstracts to assess their relevance to our study question, and grossly irrelevant articles were discarded. This was level-one screening and was done by the same two authors, FS and DM. Compiled search results of level-one screening were then searched for their full-text articles from which eligibility for inclusion was sought. This was level-two screening, and any differences of thoughts in the search process were settled by ZQ and TX. The study search, screening, and selection process are summarized in Figure 1.

### 2.5. Data Extraction

Data from included articles were independently extracted by two authors, namely, FS and DM. Disagreements were solved through discussion with ZQ, TX, and LH. Data derived from each of the included studies were study title, author name, and year of publication; study design; comparison group (cobalt chromium rods versus titanium rods); study participants' demographics including mean ages and sex; participants' number; surgical technique; mean and standard deviation of the follow-up time and clinical outcomes (correction rate, thoracic kyphosis, lumbar lordosis, rod fracture, rod fatigue life, and proximal junctional kyphosis (PJK))

### 2.6. Quality Assessment

The quality assessment of the 11 eligible studies with their design, content, and ease of use in the interpretation of results was done by using the Newcastle-Ottawa scale (NOS). The assessment was done independently by two authors, FS and DM. The disagreement was solved through discussion with third, fourth, and fifth authors, ZQ, TX, and LH. All included studies scored more than 7 stars on the Newcastle-Ottawa scale (NOS). The score indicated that the quality of all included studies was good as defined by a scale “good quality: 3 or 4 stars in selection domain, 1 or 2 stars in comparability domain, and 2 or 3 stars in outcome/exposure domain”. The results for the NOS score for each study are summarized in Table 1.

### 2.7. Statistical Analysis

Data were analyzed separately according to individual components and then it gave rise to five separate analyses: comparison of cobalt chromium and titanium rods in sagittal correction (correction rate, thoracic kyphosis, and lumbar lordosis), rod fracture, rod fatigue life, bending stiffness, and proximal junctional kyphosis (PJK). The mean difference was used to measure and compare the overall effect modified by the two-rod constructs. Forest-plots diagrammatically illustrated the overall effect. The Review Manager software (RevMan 5.3, the Cochrane Collaboration, Oxford, UK) was used to perform statistical analysis and generation of forest plots. Continuous variables were reported as mean difference (MD), standard deviation (SD) and 95% confidence interval (95% CI), while dichotomous data were presented as odds ratio (OR) and 95% confidence interval (95% CI). The software was customized to a random or fixed effect model depending on the heterogeneity (*I*^2^) of the studies when analyzing the outcomes. The fixed effect model was used when heterogeneity was less than 50%, and the random effect model was used when heterogeneity was more than 50%. Collected data were entered into the computer and rechecked by two authors, FS and DM. Some articles gave raw data; to get the data we need, we computed the mean and standard deviation by using Statistical Package for the Social Sciences (SPSS) version 25.

### 2.8. Assumptions and Simplifications

All participants, despite the study country's economic status and technological differences, were considered to have received standard surgical care.

## 3. Results

### 3.1. Study Selection

A total of fifteen full-text studies were identified through database searching for eligibility assessment. Four of these were excluded due to various reasons; Etemadifar et al. [26] compared our outcome of interest but it was randomized clinical trial rather than observational or biomechanical study; Smith et al. [27] assessed the risk factors for rod fracture and not rod fracture as an outcome; Slivka et al. [28] did not compare the outcome of our interest; Serhan et al. [29] was excluded because it did not assess the outcome of our interest. A total of eleven studies met the criteria for inclusion and included in our study.

### 3.2. Study Characteristics

The summary of the study characteristics of our eleven studies that were eligible for inclusion in our study is given in Table 2. The total number of participants with respect to the study type was 641 for observational studies and 35 for biomechanical studies. Regarding participants demographics, while observational studies recorded age and follow-up time and recruited both genders equally, biomechanical studies did not because are not applicable but were included because our study focuses only on the effectiveness of surgical rods to correct spinal deformity regardless of age, gender, or follow-up time. All observational studies reported a participant's age central tendencies by mean. While other studies used a larger sample size, others used smaller sample sizes.

Five were cohort observational studies; one was a case-control observational study; one was case series observational study, and four were biomechanical studies. Studies were conducted in different countries across continents that increase the external validity of the study. Four studies were done in USA [21, 23–25], two studies in France [12, 16], two studies in South Korea [17, 18], two studies in Japan [19, 22], and one study in Malaysia [20].

Even though the search was not set to any specified range of dates, all included studies were published after the year 2010. These studies reported the different outcomes of interest concerning our study questions. Four studies compared correction rate [12, 16, 19, 20]; five studies compared rod fracture [17–19, 24, 25]; three studies compared thoracic kyphosis [12, 16, 20], proximal junctional kyphosis [12, 17, 18], and fatigue life [21–23] each; two studies compared lumbar lordosis [12, 16] and bending stiffness [22, 23] each. Studies comparing similar outcomes were analyzed together in the same forest plot.

### 3.3. Sources of Bias

Eligible studies were assessed for risk of bias in two levels, at study level and the review level. The bias assessment at the study level shows that studies used different sample sizes. Some included studies used a large sample size, while others had a small sample size. Even though none of them reported to have calculated the required sample size before their conduction, the smaller the sample size, the less representative of the general population.

All studies compared cobalt chromium rods versus titanium rods with respect to our question of interest. Confounding factors might have been introduced to our research as factors such as gender, mean age, BMI, rod diameter, implant density, follow-up time, location of rod fracture, required bending angle, influence of rod contouring on rod strength, and stiffness were reported to some studies while other studies did not. All studies utilized posterior spinal fusion but with different constructs and maneuvers; some studies (observational studies) used patients. In contrast, others (biomechanical studies) used lumbar vertebrectomy models, but all of these studies reported our question of interest even though they use different designs. Different studies had different rod bending technique and used different type of rod bender as well as introduced different bending angles. These unusual conditions across every included study were thought to increase heterogeneity hence influence our results.

While at the review level, the risk of biases was identified as some studies had our data of interest to be extracted; other studies need calculation to obtain the required data for analysis. The overall mean age of participants in two groups could not be calculated as median was used rather than mean in some studies [20] and not reported in some studies [21–24]. Also, the overall follow-up time could not be calculated as data were not reported in some studies [20–24]. To reduce biases, the authors firstly evaluated all eligible studies by conducting database search and data extraction from different sources. Assessment for methodological biases was done before the data extraction from eligible full-text articles. To minimize reporting biases, the PRISMA (Preferred Reporting Items for Systematic Reviews and Meta-Analyses) tool was used in the study write-up.

#### 3.3.1. Correction Rate

**Figure 2:**
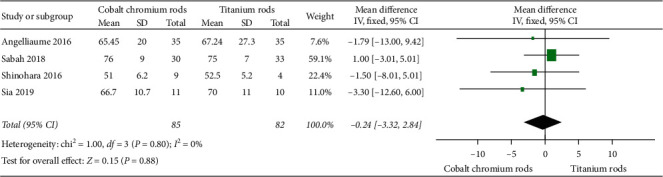
Forest plot for correction rate in percentage between cobalt chromium rods and titanium rods.

Figure 2 elucidates four [12, 16, 19, 20] of the eleven included studies that compared correction rate outcomes in percentage between cobalt chromium rods versus titanium rods. The overall mean difference between the two implants was −0.24, 95% CI (−3.32, 2.84) favouring cobalt chromium rods, but the difference did not reach statistical significance with *p* value = 0.88 (i.e. *p* value >0.05). A fixed-effect model was used since heterogeneity, *I*^2^, was 0% (i.e. *I*^2^ < 50%).

#### 3.3.2. Thoracic Kyphosis

**Figure 3:**
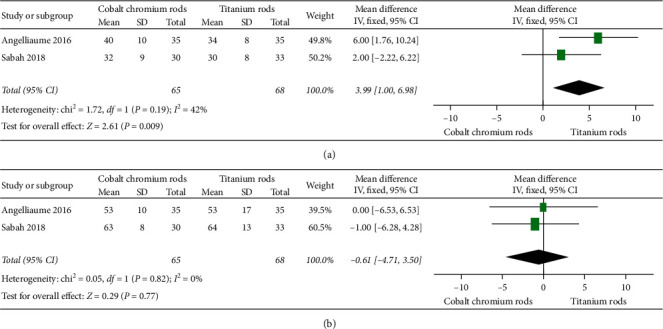
Forest plot between CoCr and Ti rods. (a) Illustrating two of eleven studies comparing thoracic kyphosis outcome results. (b) Illustrating two of eleven studies comparing lumbar lordosis outcome results.

Figure 3(a) elucidates two [12, 16] of eleven eligible studies that compared changes in thoracic kyphotic angles in degrees between cobalt chromium rods versus titanium rods. The overall mean difference between the two implants was 3.99, 95% CI (1.00, 6.98), signifying better restoration of thoracic kyphotic angle in cobalt chromium rods. The difference reached statistical significance with *p* value = 0.009 (i.e., *p* value <0.05). A fixed-effect model was used since heterogeneity, *I*^2^, was 42% (i.e. *I*^2^ < 50%).

#### 3.3.3. Lumbar Lordosis

Figure 3(b) illustrates two [12, 16] of eleven eligible studies that compared lumbar lordosis outcomes in degrees between cobalt chromium rods versus titanium rods. The overall mean difference between the two implants was −0.61, 95% CI (−4.71, 3.50) without reaching statistical significance as *p* value = 0.77 (i.e., *p* value >0.05). A fixed-effect model was used since heterogeneity, *I*^2^, was 0% (i.e. *I*^2^ < 50%).

#### 3.3.4. Rod Fracture

**Figure 4:**
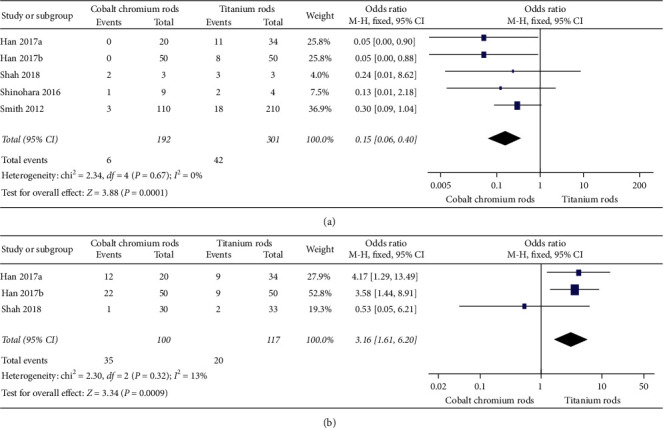
Forest plot between CoCr and Ti rods. (a) Illustrating five of eleven studies comparing rod fracture outcome results. (b) Illustrating three of eleven studies comparing proximal junctional kyphosis outcome results.

Figure 4(a) illustrates five [17–19, 24, 25] of eleven eligible studies that compared the occurrence of rod fracture between cobalt chromium rods and titanium rods. The overall odds ratio between the two implants was 0.15, 95% CI (0.06, 0.40), signifying a higher occurrence rate of rod fracture in titanium rods than that of cobalt chromium rods. The difference reached statistical significance with *p* value = 0.0001 (i.e., *p* value <0.05). A fixed-effect model was used since heterogeneity, *I*^2^, was 0% (i.e., *I*^2^ < 50%).

#### 3.3.5. Proximal Junctional Kyphosis

Figure 4(b) illustrates three [12, 17, 18] of eleven eligible studies that compared the rate of occurrence of proximal junctional kyphosis between cobalt chromium rods versus titanium rods. The odds ratio between the two implants was 3.16, 95% CI (1.61, 6.20), signifying a higher occurrence rate in cobalt chromium rods. The difference reached statistical significance with *p* value = 0.0009 (i.e., *p* value <0.05). A fixed-effect model was used since heterogeneity, *I*^2^, was 13% (i.e., *I*^2^ < 50%).

#### 3.3.6. Fatigue of Life

**Figure 5:**
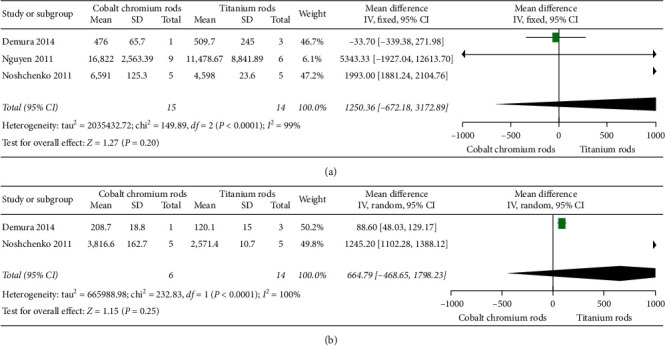
Forest plot between CoCr and Ti rods. (a) Illustrating two of eleven studies comparing fatigue of life outcome results. (b) Illustrating two of eleven studies comparing bending stiffness outcome results.

Figure 5(a) elucidates three [21–23] of eleven eligible studies that compared fatigue of life in terms of a number of loading cycles between cobalt chromium rods versus titanium rods. The overall mean difference between the two implants was 1250.36, 95% CI (−672.18, 3172.89) favouring titanium rods, but the difference did not reach statistical significance as *p* value = 0.20 (*p* value >0.05). A random effect model was used since heterogeneity was high, *I*^2^ = 99% (i.e., *I*^2^ > 50%).

#### 3.3.7. Bending Stiffness

Figure 5(b) elucidates two [22, 23] of eleven eligible studies that compared bending stiffness outcomes in terms of newtons per millimetre between cobalt chromium rods versus titanium rods. The overall mean difference between the two implants was 664.79, 95% CI (−468.65, 1798.23) without reaching statistical significance as *p* value = 0.25 (i.e., *p* value >0.05). A random effect model was used since heterogeneity was very high, *I*^2^, was 100% (i.e. *I*^2^ > 50%).

### 3.4. Sensitivity Analysis

Excluding one study as it was a randomized clinical trial conducted in Iran [26] has an impact on the statistical significance of thoracic kyphosis outcome results; however, the outcome results for deformity correction rate and lumbar lordosis did not change the statistical significance. When included, the new results were as follows: the overall mean difference of deformity correction rate was 1.57 (−3.32, 6.47), *p* value = 0.53, and *I*^2^ = 64%; the overall mean difference of thoracic kyphosis was 1.77 (−-3.09, 6.62), *p* value = 0.48, and *I*^2^ = 84%; the overall mean difference of lumbar lordosis was −0.85 (−3.30, 1.61), *p* value = 0.50, and *I*^2^ = 0%.

## 4. Discussion

The effectiveness of spinal rods after posterior instrumentation is influenced by several factors, including the body weight (BMI), rod diameter, fusion levels (implant density), rod contouring, required bending angle, and type of bender used. Biomechanical properties of rods determine their ability to correct spinal deformity and to prevent postoperative sagittal changes. Stainless steel with various diameters was previously commonly used but recently replaced by titanium rods and cobalt chromium rods [16]. The cobalt chromium gained popularity for correction of spinal deformity due to its higher mechanical strength hence less likely to have correction loss after surgery [26]. This study aimed to compare the effectiveness of cobalt chromium rods with titanium rods, in terms of their deformity correction rate; the occurrence of rod fracture; changes in thoracic kyphosis; changes in lumbar lordosis; and fatigue life of contoured rods and bending stiffness of rods, by systematic review and meta-analysis of available published literature.

The results of our meta-analysis revealed no significant difference in the main curve correction rate of spinal deformity between cobalt chromium rods and titanium rods. These findings align with those reported by Etemadifar et al. [26]; in their randomized clinical trial study on 59 patients, the authors concluded that cobalt chromium rods and titanium rods provide a similar significant primary curve correction rate, but the success rate of cobalt chromium rods was superior on spinal deformity correction. Results are also consistent with those reported by Watanabe et al. [30] and Thompson et al. [31] whereby no significant difference was observed in the correction rate between two groups implanted with titanium rods or cobalt chromium rods. Serhan et al. [29], in their biomechanical study of 80 rods, elucidated that cobalt chromium rod as compared with titanium rods has the ability to exert remarkable corrective forces on spinal deformity with less risk of developing rod deformation. Furthermore, in this study, we found that the postoperative thoracic kyphosis angle was well restored with the use of cobalt chromium rods than in the titanium rod group; there was a significant difference between them (*p* value = 0.009); the results align with those reported by Etemadifar et al. [26]. On the other hand, the findings of our study showed that there is no statistical significance in changes of lumbar lordosis with a *p* value of 0.77, which align with results reported by Han et al. [17].

The stiffness of the rod is essential not only for correction of the deformity but also for the maintenance of spine stability until a complete bone fusion achieved. However, our study revealed no significant difference between the two materials used in terms of bending stiffness (*p* value = 0.25). We believe that high heterogeneity was probably due to the variation of the methodological approach during the conduction of the respective study. Our results for bending stiffness contradicts with those reported by Noshchenko et al. [23] and Demura et al. [22] that cobalt chromium rods demonstrated higher bending stiffness than titanium rods, and there was a significant difference between them. Slivka et al. [28] elucidated that the rebending of the rods has to be avoided as it is a potential increased risk of implant failure.

In our study, we also found that the incidence of rod fracture is lower in cobalt chromium than that in titanium with a significant difference (*p* value = 0.0001), which aligns with those reported by Smith et al. [27], Han et al. [17], Shah et al. [24], Han et al. [18], and Shinohara et al. [19]. The follow-up duration for the occurrence of rod fracture is among important factors to be considered as revealed by Han et al. [17], whereby it was longer in the titanium group (56.4 ± 39.8 months) than that in the cobalt chromium group (14.0 ± 3.3 months). This means that the longer the follow-up time in the titanium group would likely show a higher incidence of rod fracture than the cobalt chromium group.

Moreover, from our study, the proximal junctional kyphosis occurs more in the cobalt chromium group with a significant difference (*p* value = 0.0009). These findings concur with the previous literature by Han et al. [17] and Han et al. [18] who reported a higher incidence of PJK in the cobalt chromium group than in the titanium group. The authors also showed a significant difference between the two groups in terms of the meantime of PJK occurrence after the operation. They reported a mean time of 3.6 months (range, 2–7 months, median: 3 months) in the cobalt chromium group and 26.3 months (range, 2–84 months, median: 24 months) in the titanium group; therefore, the follow-up duration has to be considered for the postoperative occurrence of PJK. Regarding fatigue of life, our study found that there is no statistical significance on the fatigue of life between contoured cobalt chromium rods and titanium rods (*p* value = 0.20). This result contradicts with those of Nguyen et al. [21] who concluded that the fatigue life of contoured CoCr rods is greater than that of Ti rods. The results of our meta-analysis suggest that the fatigue of life of an implant may vary due to material type used and loading condition.

Our study has some limitations, including the analysis of different studies with different designs and sample sizes; also, none of these eleven studies reported to have calculated the required sample size before their conduction. There was variability among studies concerning surgical technique as well as variability in terms of follow-up duration among included studies. The excluded randomized trial study had an impact on the statistical significance of thoracic kyphosis outcome results when included in our study but did not change the statistical significance of deformity correction rate and lumbar lordosis. Despite the presence of proximal junctional kyphosis in cobalt chromium rods, the authors of this study call upon a larger series and extensive research on the occurrence of PJK on CoCr rods to further assess its impact as a mechanical risk factor.

## 5. Conclusion

In conclusion, the cobalt chromium rod is better than a titanium rod to enhance correction of the spinal deformity as its uses increase the effectiveness and stability of the postoperative spine in a coronal and sagittal plane. However, the use of cobalt chromium rods is associated with increased risk of proximal junctional kyphosis.

## Figures and Tables

**Table 1:** Newcastle-Ottawa scale for quality assessment of the eligible studies.

Study name	Selection	Comparability	Outcome	Total score
Sabah et al. 2018 [12]	4	2	3	9
Angelliaume et al. 2016 [16]	4	2	3	9
Han et al. 2017 [17]	4	2	3	9
Han et al. 2017 [18]	4	2	3	9
Shinohara et al. 2016 [19]	4	1	3	8
Sia et al. 2019 [20]	4	1	3	8
Nguyen et al. 2011 [21]	3	2	2	7
Demura et al. 2014 [22]	3	2	2	7
Noshchenko et al. 2011 [23]	4	1	2	7
Shah et al. 2018 [24]	3	2	2	7
Smith et al. 2012 [25]	4	2	2	8

**Table 2:** Characteristics of included studies.

Study name, year	Study type	Comparison group	Number of participants	Mean age (years)	Sex F/M	Follow-up time (months)	Surgical technique	Country of study	Outcome
Sabah et al. 2018 [12]	Retrospective cohort study	CoCr	30	15 ± 2	27/3	45 ± 24	PSF (PS + ST2R)	France	CR, TK, LL, and PJK
		Ti	33	15 ± 2	27/6	41 ± 9			
Angelliaume et al. 2016 [16]	Retrospective cohort study	Ti	35	16.6 ± 4	28/7	53 ± 5	PSF (HC + PTT)	France	CR, TK and LL
		CoCr	35	15.7 ± 2	31/4	49 ± 6			
Han et al. 2017 [17]	Retrospective case-control study	CoCr	20	67.7 (55–74)	19/1	13.7 (12–23)	PSF (PS + Rods)	South Korea	PJK and RF
		Ti	34	64.8 (50–77)	33/1	56.4 (14–168			
Han et al. 2017b [18]	Retrospective cohort study	CoCr	50	69 (22–89)	36/14	25 (25–51)	PSF (PS + Rods)	South Korea	RF and PJK
		Ti	50	67 (30–79)	31/19	28.5 (24–110)			
Shinohara et al. 2016 [19]	Retrospective cohort study	Ti	4	6.7 (5–9)	12/1	68.5 (59–72)	PSF (DGRT)	Japan	CR, RF
		CoCr	9	13.8		26.1 (20–39)			
Sia et al. 2019 [20]	Prospective case series	Ti	10	16	NR	NR	PSF (PS + Rods)	Malaysia	CR and TK
		CoCr	11	17					
Nguyen et al. 2011 [21]	Biomechanical study	Ti	6	NA	NA	NA	LBVM	USA	FL
		CoCr	9						
Demura et al. 2014 [22]	Biomechanical study	CoCr	1	NA	NA	NA	TPBT	Japan	BS and FL
		Ti	3						
Igarashi et al. 2011 [23]	Biomechanical study	CoCr	5	NA	NA	NA	TPBT	USA	BS and FL
			Ti	5					
Burger et al. 2018 [24]	Biomechanical study	CoCr	3	NA	NA	NA	PSOM	USA	RF
		Ti	3						
Smith et al. 2012 [25]	Retrospective review	CoCr	110	NR	NR	NR	PSO	USA	RF
		Ti	210						

CoCr = cobalt chromium, Ti = titanium, PSF = posterior spinal fusion, PS = pedicle screw, ST2R = simultaneous translation on two rods maneuver, HC = hybrid constructs, PTT = posteromedial translation technique, CR = correction rate, TK = thoracic kyphosis, LL = lumbar lordosis, PJK = proximal junctional kyphosis, DGRT = dual growing rod technique, RF = rod fracture, FL = fatigue life, LVM = lumbar vertebrectomy models, BS = bending stiffness, TPBT = three-point bending test, PSO = pedicle subtraction osteotomy, PSOM = pedicle subtraction osteotomy models, NA = not applicable, NR = not recorded.

## Data Availability

The data supporting this study are from the previously reported studies and datasets, which have been cited. The processed data are available from the corresponding author upon request.
